# Genome-wide association study identified new susceptible genetic variants in HLA class I region for hepatitis B virus-related hepatocellular carcinoma

**DOI:** 10.1038/s41598-018-26217-7

**Published:** 2018-05-21

**Authors:** Hiromi Sawai, Nao Nishida, Seik-Soon Khor, Masao Honda, Masaya Sugiyama, Natsumi Baba, Kayoko Yamada, Norie Sawada, Shoichiro Tsugane, Kazuhiko Koike, Yuji Kondo, Hiroshi Yatsuhashi, Shinya Nagaoka, Akinobu Taketomi, Moto Fukai, Masayuki Kurosaki, Namiki Izumi, Jong-Hon Kang, Kazumoto Murata, Keisuke Hino, Sohji Nishina, Akihiro Matsumoto, Eiji Tanaka, Naoya Sakamoto, Koji Ogawa, Kazuhide Yamamoto, Akihiro Tamori, Osamu Yokosuka, Tatsuo Kanda, Isao Sakaida, Yoshito Itoh, Yuichiro Eguchi, Satoshi Oeda, Satoshi Mochida, Man-Fung Yuen, Wai-Kay Seto, Yong Poovorawan, Nawarat Posuwan, Masashi Mizokami, Katsushi Tokunaga

**Affiliations:** 1 Department of Human Genetics, Graduate School of Medicine, The University of Tokyo, Tokyo, Japan; 2 Genome Medical Science Project, National Center for Global Health and Medicine, Ichikawa, Japan; 3 Department of Gastroenterology, Kanazawa University Graduate School of Medicine, Kanazawa, Japan; 4 Epidemiology and Prevention Group, Center for Public Health Sciences, National Cancer Center, Tokyo, Japan; 5 Department of Gastroenterology, Graduate School of Medicine, The University of Tokyo, Tokyo, Japan; 6 Clinical Research Center, National Nagasaki Medical Center, Nagasaki, Japan; 7 Department of Gastroenterological Surgery I, Hokkaido University Graduate School of Medicine, Sapporo, Japan; 8 Division of Gastroenterology and Hepatology, Musashino Red Cross Hospital, Tokyo, Japan; 9 Center for Gastroenterology, Teine Keijinkai Hospital, Sapporo, Japan; 10 Department of Gastroenterology, Graduate School of Medical Sciences, International University of Health and Welfare, Narita, Japan; 11 Department of Hepatology and Pancreatology, Kawasaki Medical School, Kurashiki, Japan; 12 Department of Medicine, Shinshu University School of Medicine, Matsumoto, Japan; 13 Department of Gastroenterology and Hepatology, Hokkaido University Faculty of Medicine, Sapporo, Japan; 14 Department of Gastroenterology and Hepatology, Okayama University Graduate School of Medicine, Dentistry, and Pharmaceutical Sciences, Okayama, Japan; 15 Department of Hepatology, Osaka City University Graduate School of Medicine, Osaka, Japan; 16 Department of Gastroenterology and Nephrology, Graduate School of Medicine, Chiba University, Chiba, Japan; 17 Gastroenterology and Hepatology, Yamaguchi University Graduate School of Medicine, Yamaguchi, Japan; 18 Molecular Gastroenterology and Hepatology, Kyoto Prefectural University of Medicine, Kyoto, Japan; 19 Liver center, Saga University Hospital, Saga, Japan; 20 Division of Gastroenterology and Hepatology, Saitama Medical University, Saitama, Japan; 21 Department of Medicine and State Key Laboratory for Liver Research, The University of Hong Kong, Hong Kong, China; 22 Center of Excellence in Clinical Virology, Department of Pediatrics, Faculty of Medicine, Chulalongkorn University, Bangkok, Thailand

## Abstract

We have performed a genome-wide association study (GWAS) including 473 Japanese HBV (hepatitis B virus)-positive HCC (hepatocellular carcinoma) patients and 516 HBV carriers including chronic hepatitis and asymptomatic carrier individuals to identify new host genetic factors associated with HBV-derived HCC in Japanese and other East Asian populations. We identified 65 SNPs with P values < 10^−4^ located within the HLA class I region and three SNPs were genotyped in three independent population-based replication sets. Meta-analysis confirmed the association of the three SNPs (rs2523961: OR = 1.73, P = 7.50 × 10^−12^; rs1110446: OR = 1.79, P = 1.66 × 10^−13^; and rs3094137: OR = 1.73, P = 7.09 × 10^−9^). We then performed two-field HLA genotype imputation for six HLA loci using genotyping data to investigate the association between HLA alleles and HCC. HLA allele association testing revealed that HLA-A^*^33:03 (OR = 1.97, P = 4.58 × 10^−4^) was significantly associated with disease progression to HCC. Conditioning analysis of each of the three SNPs on the HLA class I region abolished the association of HLA-A^*^33:03 with disease progression to HCC. However, conditioning the HLA allele could not eliminate the association of the three SNPs, suggesting that additional genetic factors may exist in the HLA class I region.

## Introduction

Hepatitis B (HB) is a potentially life-threatening liver infection caused by hepatitis B virus (HBV), and approximately 248 million people worldwide are estimated to be chronically infected with HBV^1^. The clinical course of HBV infection is variable, including acute self-limiting infection, fulminant hepatic failure, inactive carrier state, and chronic hepatitis with progression to liver cirrhosis and hepatocellular carcinoma (HCC). Although some HBV carriers spontaneously eliminate the virus, every year 2–10% of individuals with chronic HB (CHB) develop liver cirrhosis, and a subset of these individuals suffer from liver failure or HCC^2^. Around 600,000 new HCC cases are diagnosed annually worldwide, and it is relatively common in Asia-Pacific countries and sub-Saharan Africa. More than 70% of HCC patients are diagnosed in Asia^3^. In contrast, HCC is relatively uncommon in the USA, Australia, and European countries^3,4^. The majority of HCC cases develop in patients with cirrhosis, which is most often attributable to chronic HBV infection followed by chronic hepatitis C virus infection in the Asia-Pacific region^5^.

Human leucocyte antigen (HLA) proteins present self and non-self peptides to T cell receptors (TCRs) to maintain self-tolerance and adapted immunity. The HLA region resides on the short arm of chromosome 6, designated as 6p21.3. It is about 3.6 Mb in length and more than 200 functional and nonfunctional genes^6,7^ are located in the region. The whole HLA region is divided into three subgroups, which are designated as class I, II, and III. The HLA class I region contains 19 HLA class I genes including 3 classical (*HLA-A*, *-B*, and -*C*), 3 non-classical (*HLA-E*, *-F*, and *-G*), and 12 non-coding genes or pseudogenes. The HLA class II region contains classical class II alpha- and beta-chain genes of *HLA-DR*, *-DQ*, and *-DP*. All HLA class I and class II molecules can present peptides to T cells, but each protein binds a different range of peptides. The presence of several different genes of each HLA class means that any one individual is equipped to present a much broader range of peptides than if only one HLA molecule of each class were expressed at the cell surface. A total of 17,695 *HLA* alleles (12,893 in class I and 4,802 in class II) were released by The IPD-IMGT/HLA database release 3.31.0 in January 2018 (https://www.ebi.ac.uk/ipd/imgt/hla/). Of the 12,893 class I alleles, 4,181, 4,950, and 3,685 alleles were registered in *HLA-A*, *-B*, and *-C* genes, respectively. Of 4,802 class II alleles, 2,146, 1,178, and 965 alleles were registered in *HLA-DRB1*, *-DQB1*, and *-DPB1* genes, respectively.

Recent genome-wide association studies (GWAS) of chronic HBV carriers with or without HCC in Chinese populations reported that one SNP (rs17401966) in *KIF1B*, two SNPs (rs9272105 and rs455804) in *HLA-DQA1/DRB1* and *GRIK1*, and two SNPs (rs7574865 and rs9275319) in *STAT4* and *HLA-DQ* were associated with disease progression to HCC^8–10^. A number of candidate genes have been investigated by genetic association studies to evaluate their roles in susceptibility to HCC. The findings from these studies, however, are inconclusive due to insufficient evidence and a lack of independent validation. All three papers referred to in this manuscript performed GWAS and replication studies using only Chinese population samples. For example, the study by Zhang *et al*.^10^ used 2,310 cases and 1,789 controls of Chinese ancestry and identified one intronic SNP in *KIF1B* associated with HBV-related HCC. This result, however, was not replicated in several other populations^11,12^). These findings suggest that GWAS and subsequent replication studies should be conducted in populations other than Chinese.

In this study, we performed GWAS using Japanese CHB patients with and without HCC and a replication study using East Asian populations including Japanese, Hong Kong Chinese, and Thai.

## Results

### GWAS and replication study of HBV-related HCC

**Figure 1:**
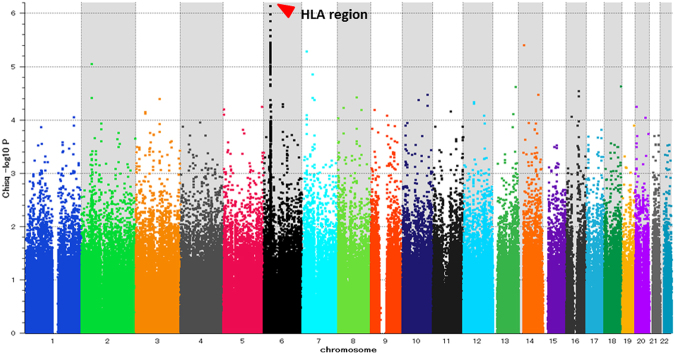
GWAS result. GWAS included 989 samples [473 Japanese HCC cases and 516 Japanese HBV carrier (CH and ASC) controls]. P-values were calculated using the chi-square test for allele frequencies among 447,830 SNPs.

We conducted a GWAS using samples from 473 Japanese HBV-positive HCC patients and 516 HBV carriers including CHB and asymptomatic carrier (ASC) individuals by analyzing 447,830 autosomal SNPs. Figure 1 shows a genome-wide view of the SNP association data based on allele frequencies. There were 110 SNPs with P values < 10^−4^ in the GWAS (Supplementary Materials, Table S1). Of the 110 SNPs, 65 and 4 SNPs were located on the HLA class I and II regions, respectively. These results suggested that HBV-related HCC could be associated with SNPs located in the HLA region, although associations did not reach the genome-wide significance level. Outside the HLA region, there were 41 SNPs with P values < 10^−4^ and 4 SNPs showed P values < 10^−5^.

In order to validate these suggestive associations, we selected seven SNPs based on the following criteria: P values < 10^−4^ in the HLA region and <10^−5^ outside the HLA region and only SNPs with the lowest P value or highest OR were selected when multiple SNPs showed strong LD. Three independent sets of HBV-related HCC cases, CHB and ASC controls (replication-1: Japanese 153 cases and 614 controls; replication-2: Hong Kong Chinese 94 cases and 187 controls; and replication-3: Thai 185 cases and 198 controls), and the original GWAS set of 989 Japanese samples (473 cases and 516 controls) were genotyped and used in a subsequent replication analysis. Of the seven SNPs, four (rs2523961, rs1110446, and rs3094137 located on HLA class I region, and rs2295119 located on HLA class II region) were validated, and consistent associations were observed between the original GWAS set and replication sets (Table 1). For these four SNPs, no heterogeneity of association was observed between the original GWAS samples and the replication samples. Two SNPs in the HLA region (rs2523961 and rs1110446) showed a genome-wide significant association (rs2523961: OR = 1.91, P = 6.42 × 10^−10^; and rs1110446: OR = 1.93, P = 2.52 × 10^−10^) using the combined Japanese samples (GWAS and replication-1) (Table 1). Moreover, the meta-analysis with the combined Japanese samples and two independent sample sets (Hong Kong Chinese and Thai) confirmed associations for the two SNPs (rs2523961: P = 5.81 × 10^−11^; and rs1110446: P = 9.09 × 10^−13^), while the remaining two SNPs showed a marginal association (rs3094137: OR = 1.76, P = 3.91 × 10^−7^; and rs2295119: OR = 0.63, P = 5.51 × 10^−7^).

**Table 1:** Four SNPs in the HLA region associated with disease progression to HCC.

Marker	Allele	stage	population	cases				controls				P value^b^	OR (95% CI)
	(1/2)			11	12	22	MAF	11	12	22	MAF		
rs2523961	A/G	GWAS	Japanese	12	174	287	0.209	11	111	394	0.129	2.57E-07	2.02 (1.54–2.66)
(class I)		Combined	Japanese	19	219	388	0.205	23	238	867	0.126	6.42E-10	1.91 (1.56–2.37)
		Replication2	Hong Kong Chinese	1	25	68	0.144	2	34	151	0.102	0.118	1.55 (0.90–2.66)
		Replication3	Thai	13	54	108	0.229	6	49	142	0.155	0.059	1.49 (0.98–2.28)
		Meta-analysis^a^										5.81E-11	
rs1110446	T/C	GWAS	Japanese	14	177	282	0.217	11	114	391	0.132	4.44E-08	2.10 (1.60–2.75)
(class I)		Combined	Japanese	21	222	383	0.211	24	245	861	0.130	2.52E-10	1.93 (1.57–2.37)
		Replication2	Hong Kong Chinese	2	22	70	0.138	1	35	151	0.099	0.138	1.52 (0.90–2.62)
		Replication3	Thai	14	66	100	0.261	5	51	142	0.154	0.002	1.93 (1.27–2.92)
		Meta-analysis^a^										9.09E-13	
rs3094137	A/G	GWAS	Japanese	9	150	314	0.178	10	97	409	0.113	9.65E-05	1.74 (1.31–2.31)
(class I)		Combined	Japanese	13	191	421	0.174	19	203	906	0.107	3.91E-07	1.76 (1.41–2.19)
		Replication2	Hong Kong Chinese	0	8	86	0.043	0	9	178	0.024	0.201	1.93 (0.71–5.21)
		Replication3	Thai	0	19	160	0.053	0	15	181	0.038	0.468	1.35 (0.60–3.03)
		Meta-analysis^a^										9.83E-05	
rs2295119	T/G	GWAS	Japanese	18	139	316	0.185	41	191	284	0.265	5.77E-06	0.59 (0.47–0.74)
(class II)		Combined	Japanese	27	179	420	0.186	78	417	635	0.254	5.51E-07	0.63 (0.53–0.76)
		Replication2	Hong Kong Chinese	2	22	70	0.138	5	54	128	0.171	0.318432	0.78 (0.47–1.28)
		Replication3	Thai	4	39	136	0.131	3	50	143	0.143	0.285443	0.76 (0.47–1.25)
		Meta-analysis^a^										4.88E-07	

^a^Results of meta-analysis were calculated by the DerSimonian-Laird method.

^b^Result of logistic regression analysis adjusted for age and sex.

### Association test for imputed HLA alleles

The two SNPs showing genome-wide significant associations were located on HLA class I region, and the marginally associated SNP was located on HLA class I and II region. To investigate the association of *HLA* alleles, we performed two-field HLA genotype imputation for six HLA loci (*HLA-A, -B, -C, -DRB1, -DQB1*, and -*DPB1*) using 989 genome-wide genotyping data used for the GWAS. Imputed *HLA* alleles were filtered (Call Threshold < 0.5) before performing association analysis for each HLA locus. The results of association tests in *HLA-A, -B, -C, -DRB1, -DQB1*, and -*DPB1* alleles are shown in Table 2 and Supplementary Materials, Table S2. To avoid false-positive results due to multiple testing for 77 HLA alleles, significance levels were set at 0.000649 (=0.05/77). A protective effect of HLA-DPB1^*^02:01 (OR = 0.59, P = 5.23 × 10^−6^) was observed as previously reported^13^. We also detected that HLA-A^*^33:03 was significantly associated with disease progression to HCC (OR = 1.97, P = 4.58 × 10^−4^) (Table 2).

**Table 2:** Association analyses of *HLA-A* alleles.

HLA-A	Case (2n = 892)	%	Control (2n = 998)	%	Fisher’s P-value	OR	95% CI
02:01	105	11.8	113	11.3	0.7733	1.04	0.78–1.40
02:06	80	9.0	106	10.6	0.2462	0.83	0.60–1.14
02:07	38	4.3	40	4.0	0.8174	1.07	0.66–1.72
11:01	53	5.9	94	9.4	0.005757	0.61	0.42–0.87
24:02	331	37.1	393	39.4	0.3198	0.91	0.75–1.10
26:01	72	8.1	89	8.9	0.5636	0.90	0.64–1.26
26:03	18	2.0	22	2.2	0.8732	0.91	0.46–1.80
31:01	112	12.6	90	9.0	0.01384	1.45	1.07–1.97
**33:03**	76	8.5	45	4.5	**0.00046**	**1.97**	1.33–2.95

Using GTEx-generated eQTL data^14^, we checked for correlations between the three SNPs and *HLA-A* gene expression levels. The SNP rs2523961 was correlated with *HLA-A* gene expression in various tissues (muscle: P = 6.1 × 10^−20^; heart: P = 2.3 × 10^−15^, 2.1 × 10^−11^; esophagus: P = 2.8 × 10^−12^, 1.8 × 10^−6^; artery: P = 4.7 × 10^−12^, 3.9 × 10^−11^; thyroid: P = 1.4 × 10^−11^; pancreas: P = 3.3 × 10^−9^; brain: P = 1.9 × 10^−8^, 2.2 × 10^−7^; nerve: P = 3.2 × 10^−8^; testis: P = 5.5 × 10^−7^; lung: P = 1.7 × 10^−5^). The SNP rs1110446 was also associated with *HLA-A* gene expression in muscle (P = 5.5 × 10^−15^), skin (P = 6.2 × 10^−11^, 4.4 × 10^−9^), artery (P = 8.7 × 10^−6^, 1.1 × 10^−4^), esophagus (P = 2.5 × 10^−5^), and whole blood (P = 5.1 × 10^−5^). These results suggest that these SNPs affected *HLA-A* gene expression.

**Figure 2:**
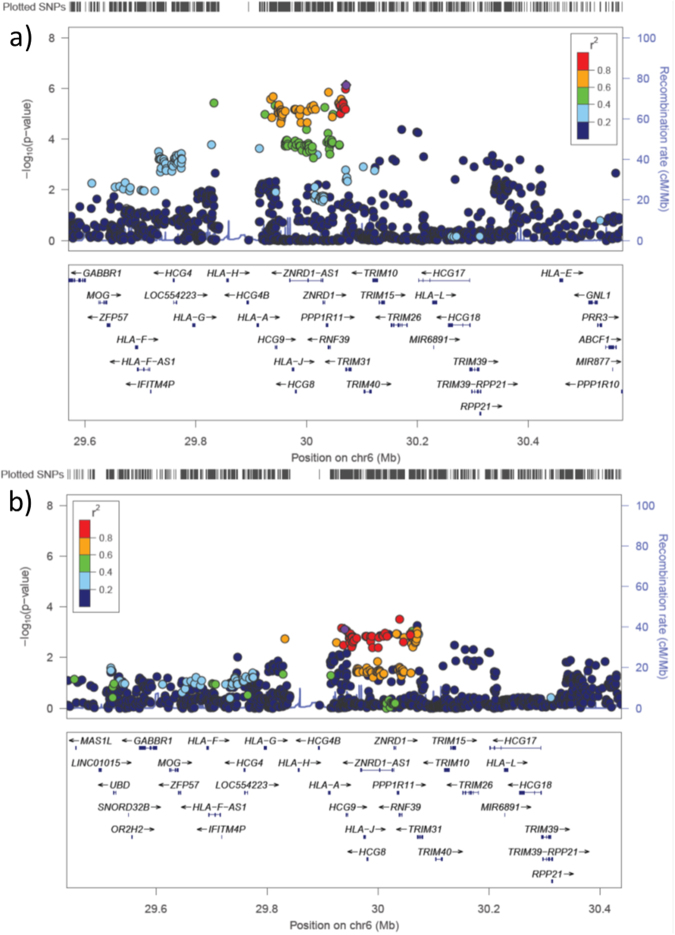
Association plots of the HLA class I region on chromosome 6 HLA region. (**a**) The major genetic determinant of HBV-related HCC risk to HLA class I genes. (**b**) Conditional analysis controlling for the effect of HLA-A*33:03.

Conditioning each of the three SNPs on the HLA class I region (Supplementary Material, Fig. S1a–c) abolished the association of HLA-A^*^33:03 (P > 0.05), but conditioning of A^*^33:03 could not eliminate the association of the three SNPs (rs2523961: OR = 1.69, P = 7.06 × 10^−4^; rs1110446: OR = 1.65, P = 9.33 × 10^−4^; and rs3094137: OR = 1.54, P = 5.68 × 10^−3^) (Fig. 2). These conditional analyses suggest that additional genetic factors other than *HLA-A* allele exist in the HLA class I region. In contrast to the class I region, conditional analysis controlling for the SNP rs2295119 using DPB1^*^02:01 allele suggests that *DPB1* allele could abolish the association of rs2295119 on the HLA class II region (P > 0.05) (Supplementary Material, Fig. S1e).

## Discussion

In the current GWAS, we found a marginal association between an SNP (rs2295119) located in the *HLA-DPB* region and HBV-related HCC. Moreover, the association analysis of *HLA-DPB1* alleles and the conditional analysis with HLA-DPB1^*^02:01 suggested that DPB1^*^02:01 was the major protective allele in the HLA class II region. Recent GWAS also showed that SNPs located in the HLA class II region (*HLA-DQA1/DRB1*^9^ and *HLA-DQ*^8^) were associated with HBV-related HCC in the Chinese population. We focused on the p-values of the HLA class II region (*HLA-DQ* and -*DR*) and six other gene regions (*KIF1B, UBE4B, PGD*, 8p12, *GRIK1* and *STAT4*) reported in previous studies and revealed the SNPs of four regions (*HLA-DQ* and -*DR*, 8p12, and *STAT4*) had p-values of less than 0.00625 (0.05/8). There were 52, 10 and 1 SNP with P < 0.00625 located on *HLA-DQ/DR*, 8p12, and *STAT4*, respectively, and the lowest p-value of each region was 0.00102 (rs9271894 on *HLA-DQA1*, OR = 1.46), 0.00278 (rs8084 on *HLA-DRA*, OR = 1.32), 0.00049 (rs13250548 on 8p12, OR = 0.68), and 0.0019 (rs6752770 on *STAT4*, OR = 1.44).

We also identified significant associations in the HLA class I region, especially around the *HLA-A* locus. The association test of imputed HLA alleles and conditional analyses with HLA-A^*^33:03 suggested that HLA-A^*^33:03 is the susceptibility allele for HCC. We performed additional conditional analyses controlling for the SNP on chromosome 6 using A^*^33:03 and DPB1^*^02:01 alleles. This indicated that *HLA-A* and *DPB1* alleles could abolish the association in the HLA class II region but were not sufficient to abolish the association in the HLA class I region (Fig. 2 and Supplementary Material, Fig. S1f). Therefore, not only the *HLA-A* allele but also additional genetic factor(s) likely exist in the HLA class I region. There are several genes in this region including *HLA-A*, *HCG9, HLA-J, HCG8, ZNRD1-AS1, ZNRD1, PPP1R11, RNF39, TRIM31*, and *TRIM40* (shown in Fig. 2). Although these genes include pseudogenes and poorly characterized genes, some are associated with various diseases. The zinc ribbon domain-containing 1 (ZNRD1) protein is associated with cell growth of gastric cancer cells^15^, angiogenesis of leukemia cells^16^, and HIV-1/AIDS disease progression^17,18^. In addition, *ZNRD1* knockdown inhibits the expression of HBV mRNA and promotes the proliferation of HepG2.2.15 cells^19^, suggesting that *ZNRD1* is one of the possible additional genetic factors at the HLA class I region. The tripartite motif-containing 31 (TRIM31) protein is essential for promoting lipopolysaccharide-induced Atg5/Atg7-independent autophagy^20^. Moreover, *TRIM40* is downregulated in gastrointestinal carcinomas and chronic inflammatory lesions of the gastrointestinal tract^21^.

Non-self antigens, such as virus-infected cells and cancer cells, and HLA class I molecules are generally recognized by the TCRs on CD8+ T lymphocytes, resulting in T cell activation^22^. The activated T cells divide and some of their progeny differentiate into lymphocytes capable of killing cells (cytotoxic T lymphocytes: CTLs) displaying the same peptides (such as tumor-specific peptides) on their HLA class I molecules. These CTLs target tumor-specific antigenic peptides and eliminate them. In other words, CTLs cannot eliminate cancer cells without HLA class I molecules even if the person has tumor-specific peptides. Cancer cells therefore need to escape from the immune system for patients to be identified as having cancer.

In this study, we identified a significant association between HLA-A^*^33:03 and HBV-related HCC. In addition to HLA-A^*^33:03, previous studies and this study suggested that *HLA-DR*, -*DQ*, and -*DP* were associated with disease progression^8,9,13^. Functional analysis of HLA class I and II proteins could be an important step in determining the pathology of HBV-related HCC.

## Methods

### Ethics statement

All study protocols conformed to the relevant ethical guidelines, as reflected in the *a priori* approval by the ethics committee of the University of Tokyo, and by the ethics committees of all participating universities and hospitals. All participating studies obtained informed consent from all participants in this study and all samples were anonymized.

### Samples

Samples from 3,133 individuals who had HBV-derived chronic hepatitis, ASC, liver cirrhosis, or HCC and patients with other HBV-related symptoms were collected by 26 universities and hospitals (Hokkaido University Hospital, Teine Keijinkai Hospital, Iwate Medical University Hospital, Musashino Red Cross Hospital, The University of Tokyo Hospital, Saitama Medical University Hospital, Chiba University Hospital, Kitasato University Hospital, Kohnodai Hospital, Shinshu University Hospital, Kanazawa University Hospital, Nagoya City University Hospital, Kyoto Prefectural University of Medicine Hospital, National Hospital Organization Osaka National Hospital, Osaka City University Hospital, Hyogo College of Medicine, Tottori University Hospital, Ehime University Hospital, Yamaguchi University Hospital, Kawasaki Medical College Hospital, Okayama University Hospital, Nagasaki Medical Center, Kurume University Hospital, Saga University Hospital, Eguchi Hospital, and Kyusyu University Hospital). The Japanese Public Health Cancer-based Prospective (JPHC) Study samples^23^ in Japan were used for the replication study. Hong Kong Chinese samples were collected at the University of Hong Kong. Thai samples were collected at Chulalongkorn University.

HBV status was measured based on serological results for HBsAg and anti-HBc with a fully automated chemiluminescent enzyme immunoassay system (Abbott ARCHITECT, Abbott Japan, Tokyo, Japan or LUMIPULSE G1200, Fujirebio, Inc., Tokyo, Japan). For clinical staging, ASC state was defined by the presence of HBsAg with normal ALT levels over 1 year (examined at least four times at 3-month intervals) and without evidence of liver cirrhosis. CH was defined by elevated ALT levels (1.5 times the upper limit of normal [35 IU/L]) persisting for over 6 months (by at least three bimonthly tests). HCC was diagnosed by ultrasonography, computerized tomography, magnetic resonance imaging, angiography, tumor biopsy, or by a combination of these.

### SNP genotyping and data cleaning

For the GWAS, we genotyped 1,356 Japanese samples using the Affymetrix Axiom Genome-Wide ASI 1 Array (Affymetrix, Inc., Santa Clara, CA, USA) according to the manufacturer’s instructions and determined the genotype calls of 600,307 SNPs using the Genotyping Console v4.2.0.26 software (Supplementary Material, Fig. S2a). To increase the samples for genotyping, we used not only CHB patients with and without HCC but also patients with HBV-related other symptoms such as liver cirrhosis. All samples used for genotyping passed a Dish QC >0.82 and overall call rate >97%. The average Dish QC for 1,356 samples was 0.969 (0.883–0.993) and the average call rate reached 99.42% (97.47–99.87%). All genotyped samples passed a heterozygosity check, and 25 duplicated samples were identified in identity by descent (IBD) testing. A principal component analysis (PCA) found seven outliers could be excluded by the Smirnov-Grubbs test, and we showed that all the remaining samples (n = 1,324) formed a single cluster with the HapMap Japanese (JPT) samples but not with the Han Chinese (CHB), Northern and Western European (CEU), and Yoruban (YRI) samples. We then applied the following thresholds for SNP quality control in data cleaning: SNP call rate of ≥95%, minor allele frequency of ≥3% and Hardy-Weinberg equilibrium P value of ≥0.001. A total of 447,830 SNPs on autosomal chromosomes passed the quality control filters and were used for subsequent GWAS. For the association study of HBV-related HCC, we selected 481 HBV-related HCC patients (cases) and 538 HBV carriers (CH and ASC patients, controls) from 1,324 samples and performed IBD testing and PCA again for these samples. Twenty-three related samples and seven outliers were excluded by IBD testing and PCA (Supplementary Material, Fig. S3), respectively. We finally used 473 cases and 516 controls for GWAS. A quantile-quantile plot of the distribution of test statistics for the comparison of genotype frequencies in the cases and controls showed that the inflation factor λ was 1.016 for all tested SNPs and was 1.009 when SNPs in the HLA region were excluded (Supplementary Material, Fig. S4). All cluster plots for SNPs with P values of <10^−4^ were checked visually and SNPs with ambiguous genotype calls were excluded.

In the replication stage, we selected seven SNPs with P values of <10^−5^ from the results of the chi-square test in the GWAS. A TaqMan SNP genotyping assay (Applied Biosystems, Foster City, CA, USA) was used to confirm the genotypes at each SNP. We genotyped 989 and 767 Japanese samples for the validation of the GWAS and for the replication study, respectively. We further genotyped 281 Hong Kong Chinese and 383 Thai samples for the replication study (Supplementary Materials, Table S3).

### Statistical analysis

The characteristics of analyzed samples are shown in Supplementary Materials, Table S3. For the GWAS and replication study, the chi-square test was applied to a two-by-two contingency table in the allele frequency model. Meta-analysis was performed using the DerSimonian-Laird method (random-effects model) in order to calculate the pooled OR and its 95% confidence interval. Fisher’s exact test in a two-by-two contingency table was used to examine the association between *HLA* alleles and disease progression of HBV patients. To avoid false-positive results due to multiple testing, the resulting P-values were adjusted based on the number of observed alleles with frequencies ≥0.5% in cases and controls. Conditional logistic regression analysis was performed for SNPs and *HLA* alleles. This analysis was performed as implemented in Plink v1.07 software^24^, conditioning on HLA-A^*^33:03 and DPB1^*^02:01 to each of the other SNPs. Other statistical analyses were performed using the SNP & Variation Suite 7 software (Golden Helix, Bozeman, MT, USA) and statistical software R v2.6. Manhattan plot of conditioning of each SNP or HLA allele was generated by LocusZoom^25^.

### HLA imputation

SNP data from 989 samples were extracted from extended MHC (xMHC) regions ranging from 25759242 bp to 33534827 bp based on hg19 position. Two-field HLA genotype imputation was performed for a total of six HLA class I and class II genes using the HIBAG R package^26,27^. For *HLA-A,-B, -DRB1, -DQB1*, and *-DPB1*, a Japanese imputation reference^26^ was used for HLA genotype imputation. For *HLA-C*, the HIBAG Asian reference^27^ was used for HLA genotype imputation. We applied post-imputation quality control using call-threshold (CT > 0.5); the call rate of successfully imputed samples ranged from 88.7 to 98.5% for the six HLA classes. In total, we imputed 5,650 HLA genotypes in HLA class I and class II genes.

## Electronic supplementary material

Supplementary Information
