# Supplementary material for: Genome-wide association study identified new susceptible genetic variants in HLA class I region for hepatitis B virus-related hepatocellular carcinoma
Source: Sci Rep. 2018 May 21;8:7958. doi: 10.1038/s41598-018-26217-7 (PMC5962604; doi:10.1038/s41598-018-26217-7)
Supplement: Supplementary file 1 — Supplementary Information [file 41598_2018_26217_MOESM1_ESM.pdf]

SUPPLEMENTARY INFORMATION

Title: Genome-wide association study identified new susceptible genetic variants in HLA class I region for hepatitis B virus-related hepatocellular carcinoma

Authors: Hiromi Sawai*, Nao Nishida, Seik-Soon Khor, Masao Honda, Masaya Sugiyama, Natsumi Baba, Kayoko Yamada, Norie Sawada, Shoichiro Tsugane, Kazuhiko Koike, Yuji Kondo, Hiroshi Yatsuhashi, Shinya Nagaoka, Akinobu Taketomi, Moto Fukai, Masayuki Kurosaki, Namiki Izumi, Jong-Hon Kang, Kazumoto Murata, Keisuke Hino, Sohji Nishina, Akihiro Matsumoto, Eiji Tanaka, Naoya Sakamoto, Koji Ogawa, Kazuhide Yamamoto, Akihiro Tamori, Osamu Yokosuka, Tatsuo Kanda, Isao Sakaida, Yoshito Itoh, Yuichiro Eguchi, Satoshi Oeda, Satoshi Mochida, Man-Fung Yuen, Wai-Kay Seto, Yong Poovorawan, Nawarat Posuwan, Masashi Mizokami and Katsushi Tokunaga

a) rs2523961

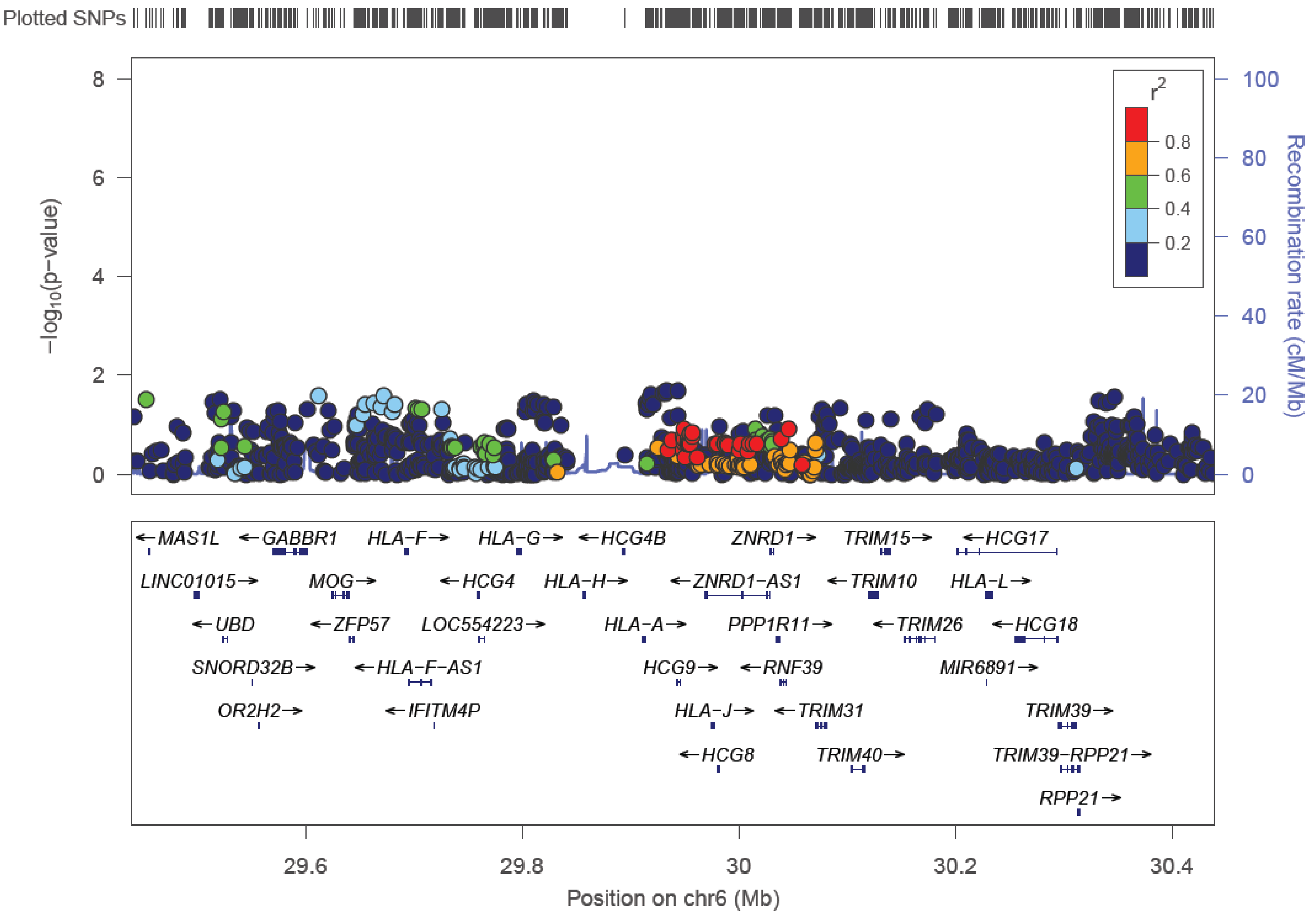

Plotted SNPs
−log10(p−value)
8
6
4
2
0
r2
0.8
0.6
0.4
0.2
100
80
60
40
20
0
Recombination rate (cM/Mb)
←MAS1L
←GABBR1
HLA−F→
HLA−G→
←HCG4B
ZNRD1→
TRIM15→
←HCG17
LINC01015→
MOG→
←HCG4
HLA−H→
←ZNRD1−AS1
←TRIM10
HLA−L→
←UBD
←ZFP57
LOC554223→
HLA−A→
PPP1R11→
←TRIM26
←HCG18
SNORD32B→
←HLA−F−AS1
HCG9→
←RNF39
MIR6891→
OR2H2→
←IFITM4P
HLA−J→
←TRIM31
TRIM39→
←HCG8
TRIM40→
TRIM39−RPP21→
RPP21→
29.6
29.8
30
30.2
30.4
Position on chr6 (Mb)

b) rs1110446

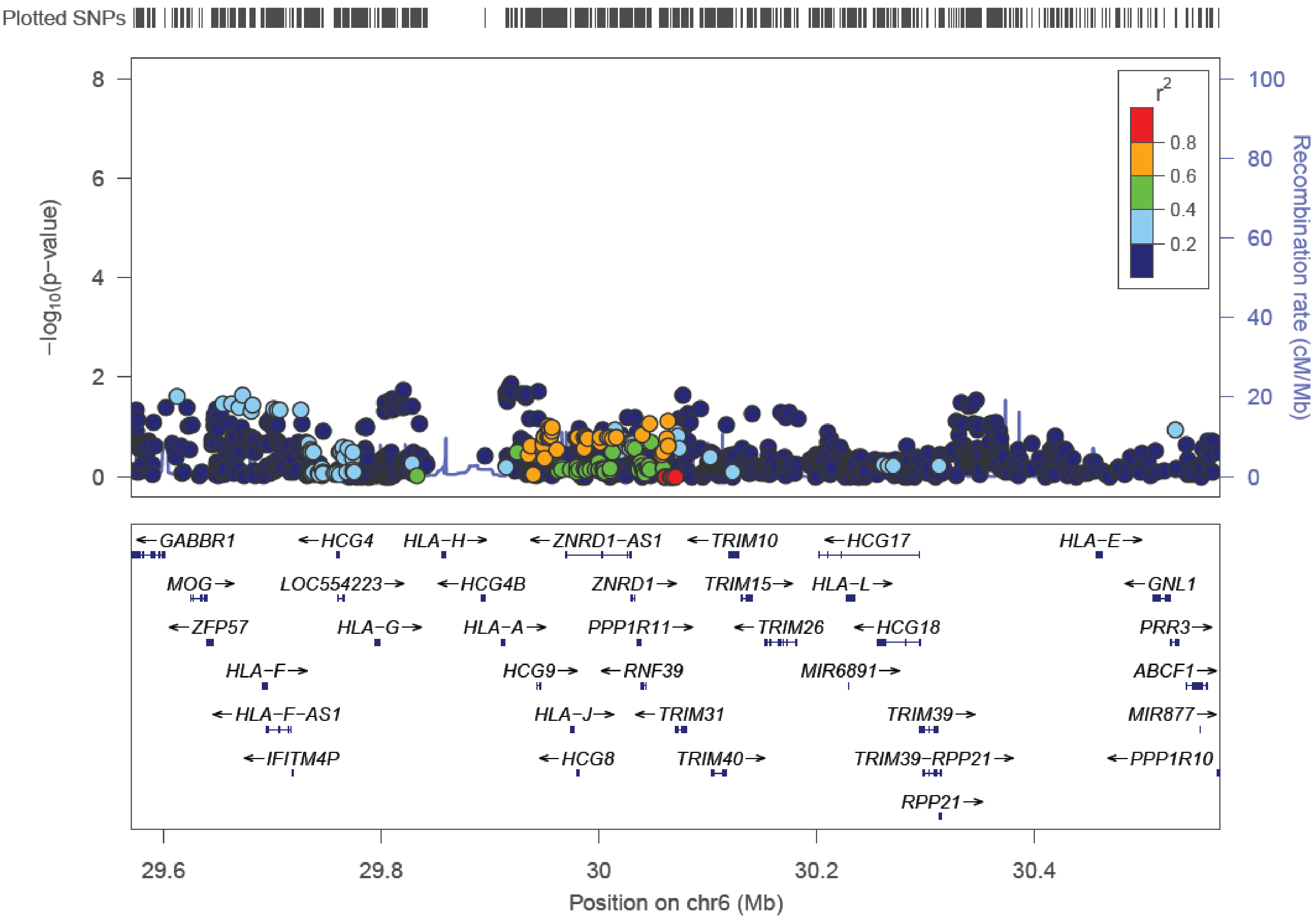

Plotted SNPs
−log10(p−value)
Recombination rate (cM/Mb)
r2
0.8
0.6
0.4
0.2
0
2
4
6
8
0
20
40
60
80
100
GABBR1
HCG4
HLA−H
ZNRD1−AS1
TRIM10
HCG17
HLA−E
MOG
LOC554223
HCG4B
ZNRD1
TRIM15
HLA−L
GNL1
ZFP57
HLA−G
HLA−A
PPP1R11
TRIM26
HCG18
PRR3
HLA−F
HCG9
RNF39
MIR6891
ABCF1
HLA−F−AS1
HLA−J
TRIM31
TRIM39
MIR877
IFITM4P
HCG8
TRIM40
TRIM39−RPP21
PPP1R10
RPP21
29.6
29.8
30
30.2
30.4
Position on chr6 (Mb)

c) rs3094137

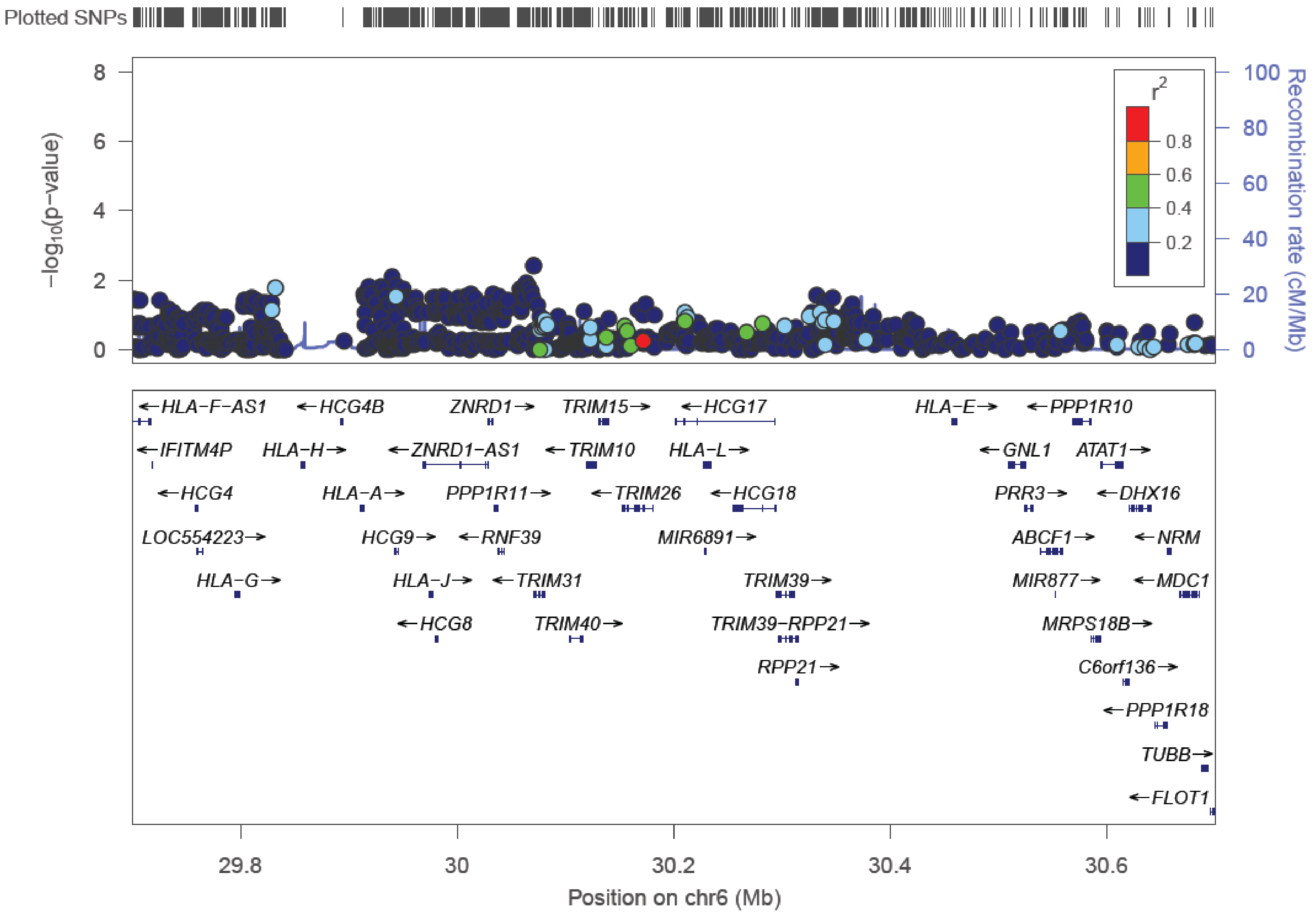
Plotted SNPs
−log10(p−value)
8
6
4
2
0
100
80
60
40
20
0
Recombination rate (cM/Mb)
r2
0.8
0.6
0.4
0.2
←HLA−F−AS1
←HCG4B
ZNRD1→
TRIM15→
←HCG17
HLA−E→
←PPP1R10
←IFITM4P
HLA−H→
←ZNRD1−AS1
←TRIM10
HLA−L→
←GNL1
ATAT1→
←HCG4
HLA−A→
PPP1R11→
←TRIM26
←HCG18
PRR3→
←DHX16
LOC554223→
HCG9→
←RNF39
MIR6891→
ABCF1→
←NRM
HLA−G→
HLA−J→
←TRIM31
TRIM39→
MIR877→
←MDC1
←HCG8
TRIM40→
TRIM39−RPP21→
MRPS18B→
RPP21→
C6orf136→
←PPP1R18
TUBB→
←FLOT1
29.8
30
30.2
30.4
30.6
Position on chr6 (Mb)

d) rs2295119

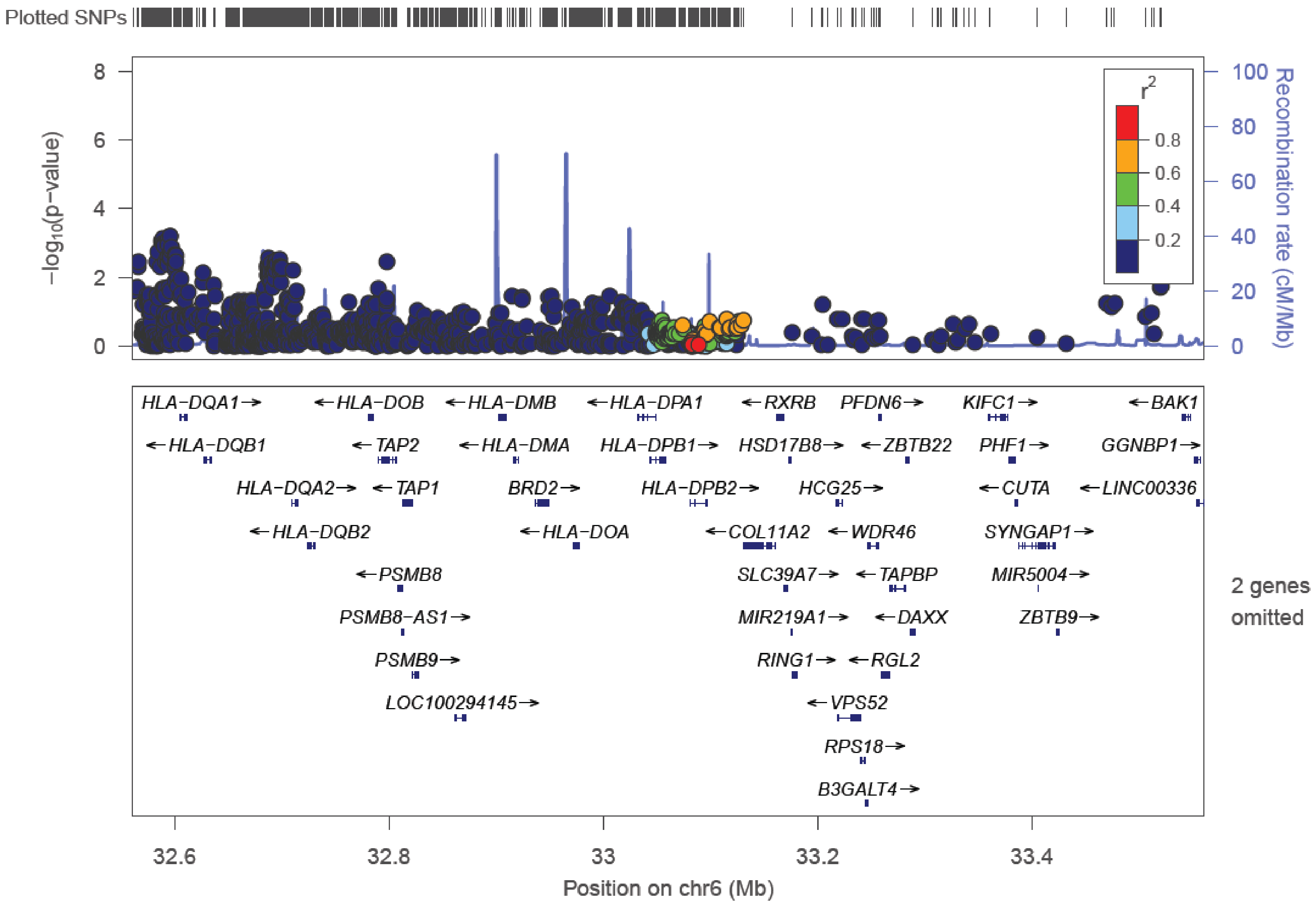

Plotted SNPs
−log10(p−value)
8
6
4
2
0
100
80
60
40
20
0
Recombination rate (cM/Mb)
r2
0.8
0.6
0.4
0.2
HLA−DQA1→
←HLA−DQB1
HLA−DQA2→
←HLA−DQB2
←HLA−DOB
←TAP2
←TAP1
←PSMB8
PSMB8−AS1→
PSMB9→
LOC100294145→
←HLA−DMB
←HLA−DMA
BRD2→
←HLA−DOA
←HLA−DPA1
HLA−DPB1→
HLA−DPB2→
←COL11A2
←RXRB
HSD17B8→
HCG25→
SLC39A7→
MIR219A1→
RING1→
←VPS52
RPS18→
B3GALT4→
PFDN6→
←ZBTB22
←WDR46
←TAPBP
←DAXX
←RGL2
KIFC1→
PHF1→
←CUTA
SYNGAP1→
MIR5004→
ZBTB9→
←BAK1
GGNBP1→
←LINC00336
2 genes omitted
32.6
32.8
33
33.2
33.4
Position on chr6 (Mb)

e) *HLA-DPB1*02:01*

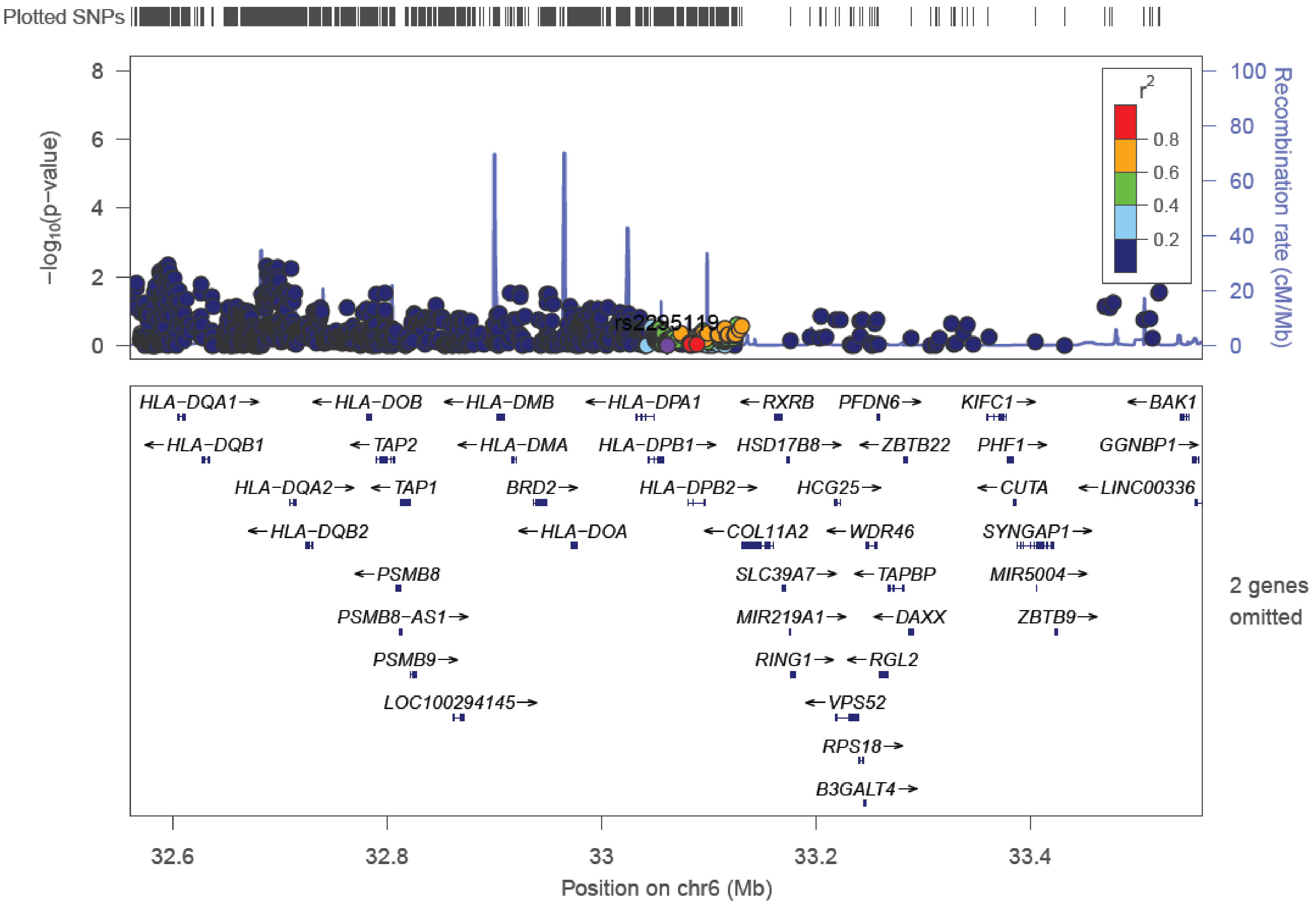

f) *HLA-A*33:03* and *DPB1*02:01*

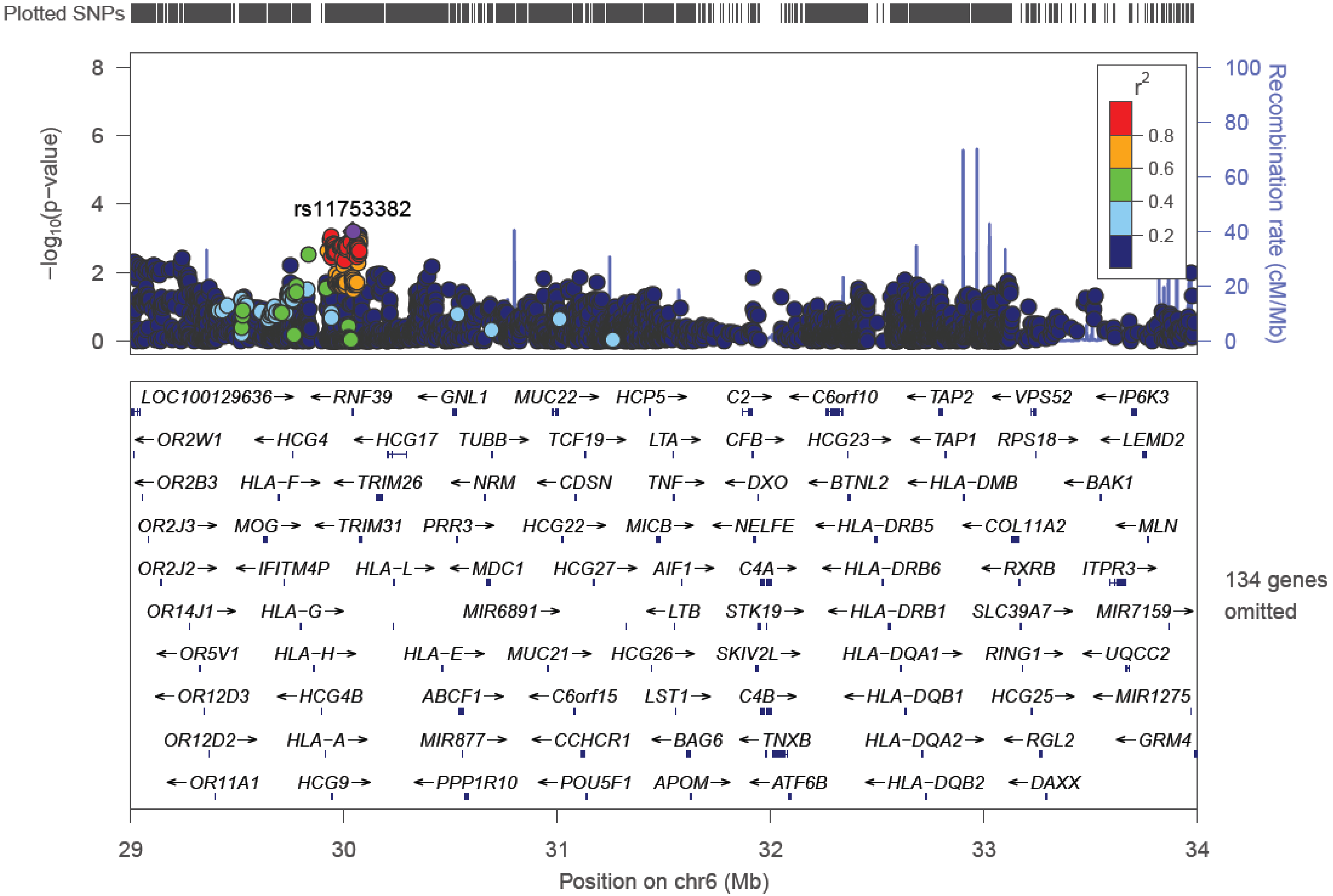

Figure S1. Association plots of chromosome 6 HLA regions

Conditional analysis controlling for the effect of a) rs2523961, b) rs1110446, c) rs3094137, d) rs2295119, e) *HLA-DPB1*02:01*, and f) *HLA-A*33:03* and *DPB1*02:01*.

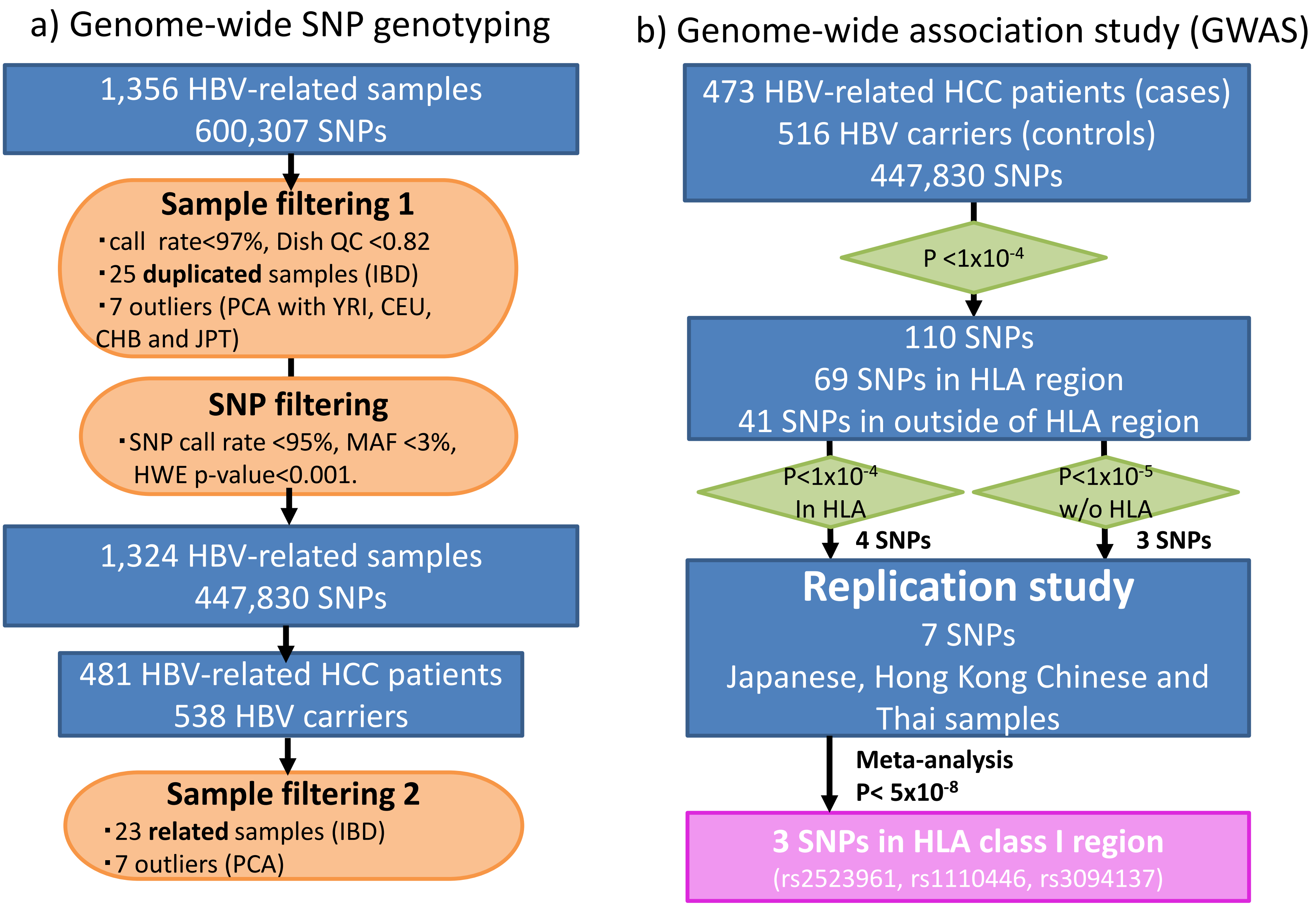

Figure S2. Flowchart describing the analysis strategy for the study

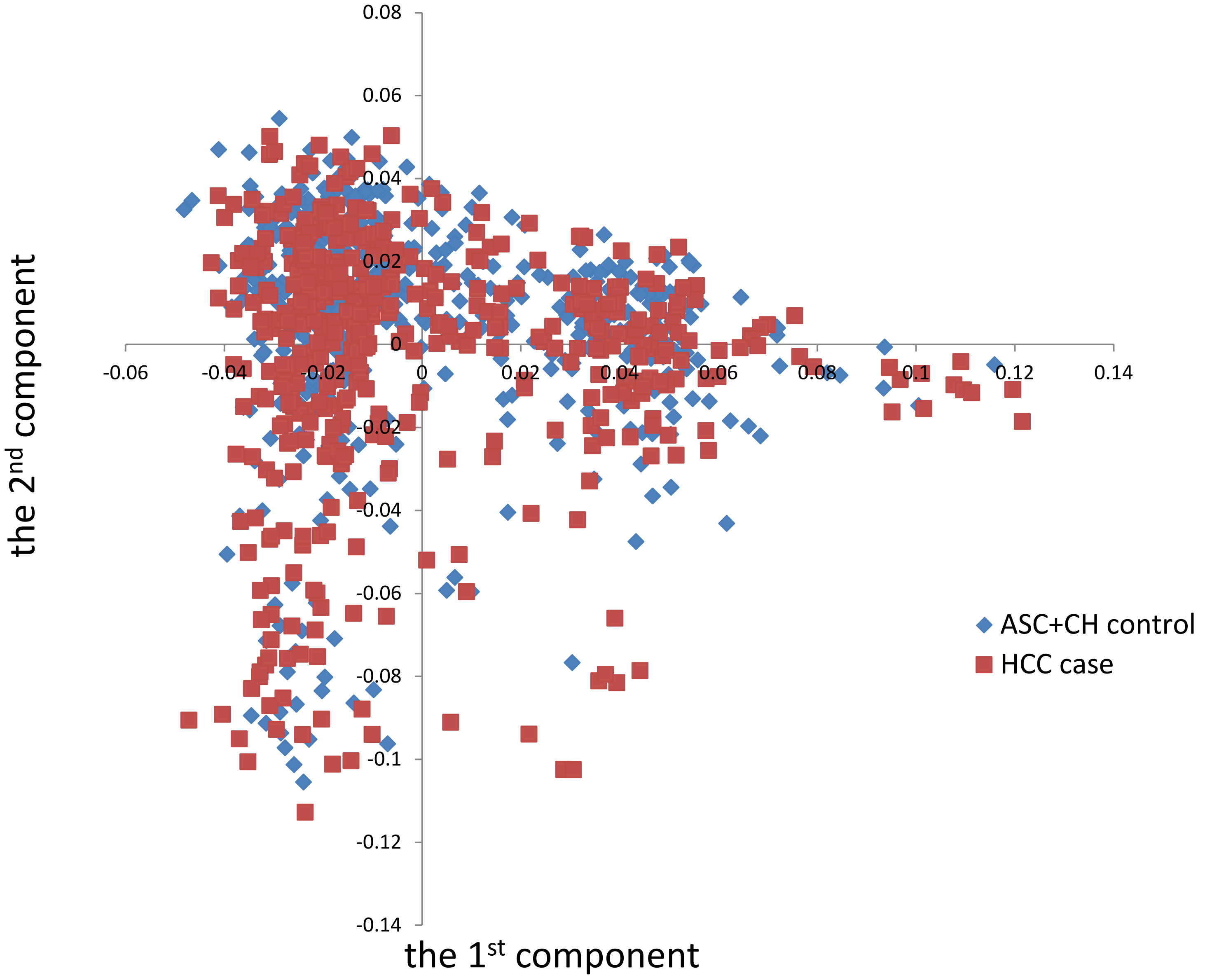

Figure S3. Principal component analysis of 989 samples from the GWAS stage

a) All the tested SNPs

b) SNPs in the HLA region were excluded

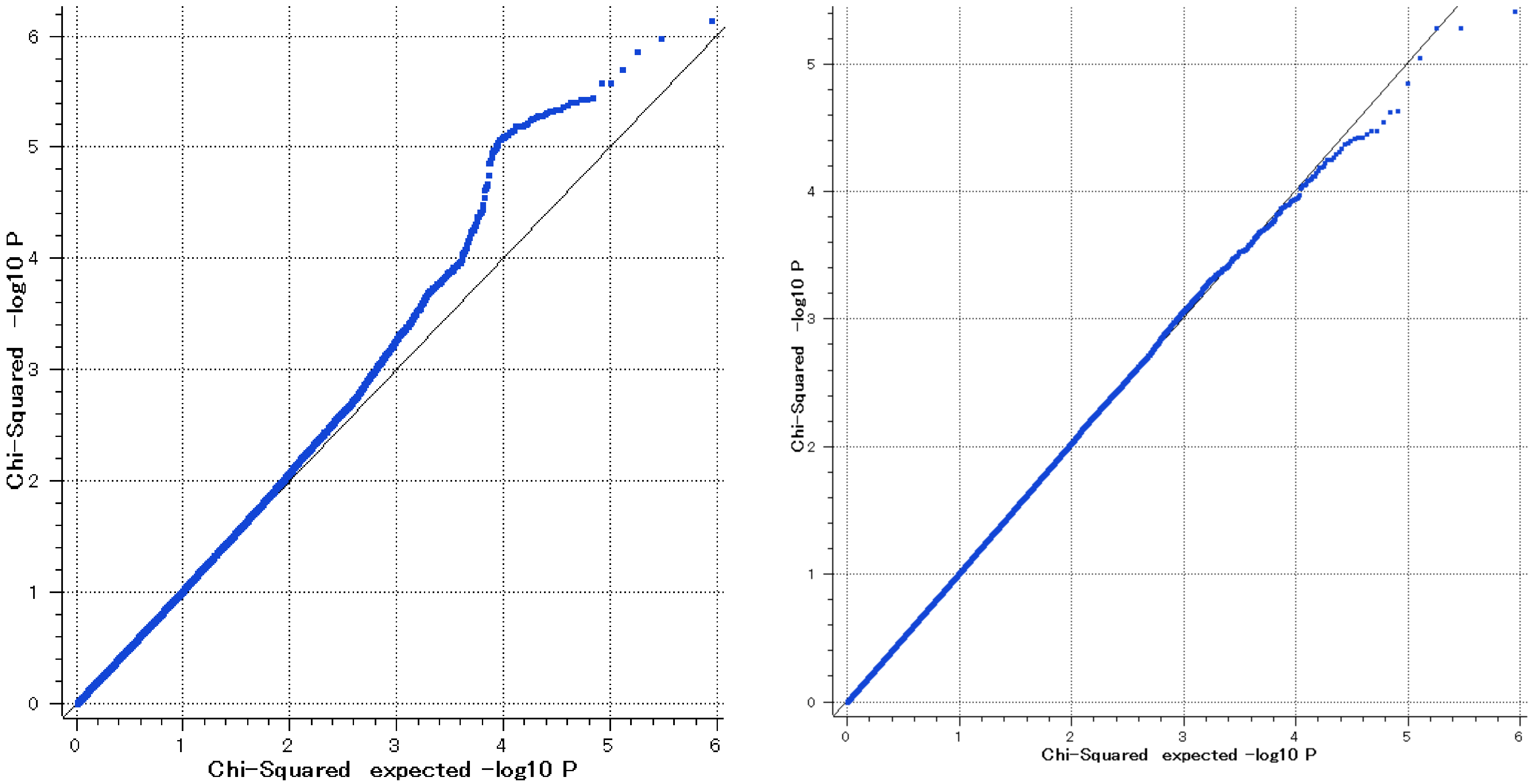

Figure S4. Quantile-quantile plot of the allele-based chi-square test statistics for the GWAS

Table S1. List of 110 SNPs with $P < 10^{-4}$ in the GWAS

Marker	Chromoso	Position	dbSNP RS ID	Associated Gene	Reference Alleles	Chi-Square P	OR[a]	95% CI Low	95% CI High	Minor Allele	MAF	Allele Freq Cases	Allele Freq Controls	HWE P (Controls	cases DD	cases Dd	cases dd	controls DD	controls Dd	controls dd
AX-11378164	1	216681000	rs2244598	ESRRG	[T/C]	8.90E-05	1.50	1.22	1.84	A	0.256	0.296	0.219	0.897	44	188	234	25	174	313
AX-11478368	2	45640334	rs3755072	SRBD1	[T/C]	3.78E-05	1.50	1.24	1.82	B	0.302	0.346	0.261	0.977	57	213	202	35	199	281
AX-12632107	2	45694417	rs7561623	SRBD1	[A/G]	8.91E-06	1.55	1.28	1.88	A	0.297	0.344	0.253	0.999	58	209	205	33	195	288
AX-12558986	3	41081808	rs369775	CTNNB1	[T/G]	7.52E-05	0.61	0.47	0.78	A	0.156	0.123	0.187	0.754	7	102	364	17	159	339
AX-12559031	3	41096041	rs371341	CTNNB1	[T/C]	7.05E-05	0.60	0.46	0.77	A	0.147	0.114	0.177	0.748	6	95	369	15	151	344
AX-12401403	3	105966369	rs10933840	LOC100302640	[T/G]	3.97E-05	1.47	1.22	1.78	A	0.349	0.395	0.307	0.786	71	232	170	50	217	249
AX-12533870	5	4256547	rs263733	LOC340094	[T/C]	7.88E-05	1.50	1.23	1.84	A	0.257	0.298	0.220	0.301	36	209	227	29	169	318
AX-11402615	5	4256610	rs263734	LOC340094	[A/G]	6.35E-05	1.51	1.23	1.85	B	0.258	0.299	0.220	0.301	36	210	226	29	169	318
AX-11427859	5	173760242	rs2936978	MSX2	[C/G]	5.61E-05	0.64	0.51	0.79	A	0.216	0.177	0.251	0.692	12	142	316	34	189	288
AX-11396793	6	29832332	rs2524035	HLA-H	[A/G]	3.68E-06	1.75	1.38	2.21	A	0.172	0.213	0.135	0.514	14	173	284	11	116	386
AX-11221788	6	29924779	rs12665039	HLA-J	[T/C]	1.04E-05	1.70	1.34	2.16	B	0.170	0.209	0.135	0.319	13	172	288	12	115	389
AX-11396327	6	29934543	rs2517677	HLA-J	[A/G]	2.59E-06	1.77	1.39	2.24	A	0.169	0.211	0.131	0.661	13	173	286	10	115	389
AX-11396326	6	29937242	rs2517673	HLA-J	[A/G]	1.39E-05	1.69	1.33	2.14	A	0.172	0.210	0.136	0.196	13	173	287	13	114	386
AX-11396780	6	29939580	rs2523961	HLA-J	[A/G]	2.02E-06	1.78	1.40	2.27	A	0.168	0.210	0.130	0.352	12	174	286	11	111	391
AX-11408202	6	29943490	rs2735076	HLA-J	[A/G]	4.70E-06	1.74	1.37	2.21	A	0.169	0.209	0.132	0.437	14	170	289	11	114	390
AX-11367732	6	29948365	rs2108691	HLA-J	[T/G]	6.16E-06	1.73	1.36	2.19	A	0.169	0.209	0.133	0.466	12	173	286	11	115	390
AX-11408201	6	29948482	rs2735069	HLA-J	[T/G]	8.50E-06	1.72	1.35	2.18	A	0.168	0.207	0.132	0.441	11	173	286	11	114	389
AX-11497582	6	29949123	rs423808	HLA-J	[T/G]	5.56E-06	1.73	1.36	2.20	A	0.169	0.209	0.132	0.437	12	173	287	11	114	390
AX-11490714	6	29949168	rs410909	HLA-J	[T/G]	7.29E-06	1.72	1.36	2.19	A	0.168	0.208	0.132	0.437	11	174	287	11	114	390
AX-11396345	6	29949293	rs2517809	HLA-J	[A/G]	9.50E-06	1.71	1.35	2.17	A	0.170	0.209	0.134	0.510	12	172	284	11	116	387
AX-11312714	6	29949456	rs17180542	HLA-J	[T/C]	5.21E-06	1.74	1.37	2.21	A	0.168	0.208	0.131	0.405	12	171	286	11	113	391
AX-11480967	6	29950140	rs379221	HLA-J	[A/G]	4.28E-06	1.74	1.37	2.21	B	0.169	0.209	0.132	0.433	12	174	287	11	114	391
AX-11313017	6	29952704	rs17186930	HLA-J	[T/C]	2.26E-05	1.68	1.32	2.13	A	0.167	0.204	0.133	0.446	11	170	290	11	114	388
AX-11313018	6	29952814	rs17186937	HLA-J	[T/C]	7.19E-06	1.73	1.36	2.19	B	0.167	0.206	0.131	0.401	12	171	290	11	113	392
AX-11313019	6	29952920	rs17186944	HLA-J	[T/C]	8.50E-06	1.72	1.35	2.19	A	0.166	0.206	0.131	0.401	12	170	290	11	113	392
AX-11173079	6	29953099	rs11755961	HLA-J	[T/C]	8.26E-06	1.72	1.35	2.19	A	0.166	0.206	0.131	0.382	12	170	290	11	112	390
AX-11221640	6	29954111	rs12662947	HLA-J	[T/C]	7.10E-06	1.73	1.36	2.19	B	0.167	0.207	0.131	0.405	12	171	289	11	113	391
AX-11221552	6	29954185	rs12661402	HLA-J	[T/C]	1.75E-05	1.69	1.33	2.16	A	0.164	0.201	0.129	0.585	9	171	290	10	113	391
AX-11221554	6	29954199	rs12661411	HLA-J	[T/C]	7.01E-06	1.73	1.36	2.20	A	0.168	0.207	0.131	0.409	12	171	288	11	113	390
AX-11454807	6	29954617	rs34894339	HLA-J	[A/G]	1.08E-05	1.70	1.34	2.16	B	0.169	0.207	0.133	0.137	13	170	290	13	111	391
AX-11462575	6	29954642	rs35356382	HLA-J	[T/C]	8.40E-06	1.72	1.35	2.19	A	0.167	0.206	0.131	0.405	12	170	289	11	113	391
AX-11313031	6	29955222	rs17187077	HLA-J	[A/C]	1.21E-05	1.70	1.34	2.17	B	0.166	0.204	0.131	0.405	11	171	290	11	113	391
AX-11221795	6	29956842	rs12665186	HLA-J	[A/G]	8.26E-06	1.72	1.35	2.19	B	0.166	0.206	0.131	0.382	12	170	290	11	112	390
AX-11221620	6	29957077	rs12662611	HLA-J	[T/C]	1.04E-05	1.71	1.34	2.17	A	0.168	0.207	0.132	0.248	12	171	289	12	112	390
AX-11313039	6	29961579	rs17187224	HLA-J	[T/C]	7.87E-06	1.73	1.36	2.20	A	0.163	0.202	0.128	0.826	12	167	293	9	114	393
AX-11221524	6	29978027	rs12661053	ZNRD1	[T/C]	6.49E-06	1.73	1.36	2.20	A	0.167	0.207	0.131	0.401	12	171	289	11	113	392
AX-11172899	6	29979643	rs11753431	ZNRD1	[A/G]	7.10E-06	1.73	1.36	2.19	A	0.167	0.207	0.131	0.405	12	171	289	11	113	391
AX-11172945	6	29980240	rs11753960	ZNRD1	[T/C]	6.41E-06	1.73	1.36	2.20	A	0.167	0.207	0.131	0.405	12	171	288	11	113	391
AX-11595286	6	29986142	rs6931672	ZNRD1	[A/G]	7.76E-06	1.72	1.35	2.19	A	0.167	0.207	0.131	0.409	12	171	289	11	113	390
AX-11280252	6	29986684	rs165256	ZNRD1	[T/C]	7.78E-06	1.72	1.35	2.19	B	0.168	0.207	0.132	0.437	11	173	286	11	114	390
AX-11313055	6	29987191	rs17187488	ZNRD1	[A/G]	1.11E-05	1.71	1.34	2.17	A	0.166	0.204	0.131	0.401	11	171	290	11	113	392
AX-11595078	6	29988964	rs6928966	ZNRD1	[T/C]	6.49E-06	1.73	1.36	2.20	B	0.167	0.207	0.131	0.401	12	171	289	11	113	392
AX-11133546	6	29989689	rs10947045	ZNRD1	[T/C]	2.13E-05	1.68	1.32	2.14	A	0.168	0.206	0.134	0.267	12	168	286	12	112	384
AX-11506046	6	29999534	rs4448133	ZNRD1	[A/G]	4.20E-05	1.53	1.25	1.87	B	0.269	0.312	0.229	0.711	37	213	210	25	181	298
AX-11133547	6	30000973	rs10947046	ZNRD1	[T/G]	7.86E-06	1.72	1.35	2.19	B	0.167	0.206	0.131	0.405	12	171	290	11	113	391
AX-11133548	6	30007711	rs10947047	ZNRD1	[A/G]	5.28E-06	1.74	1.37	2.21	A	0.167	0.207	0.131	0.401	12	171	287	11	113	392
AX-11221776	6	30009664	rs12664794	ZNRD1	[A/G]	6.49E-06	1.73	1.36	2.20	B	0.167	0.207	0.131	0.401	12	171	289	11	113	392
AX-11595922	6	30012340	rs6940552	ZNRD1	[A/G]	4.57E-06	1.75	1.38	2.23	A	0.166	0.206	0.129	0.562	12	171	290	10	112	389
AX-11565315	6	30016297	rs6457138	ZNRD1	[A/C]	6.49E-06	1.73	1.36	2.20	B	0.167	0.207	0.131	0.401	12	171	289	11	113	392
AX-11436714	6	30032673	rs3188482	ZNRD1	[A/G]	5.48E-06	1.74	1.37	2.21	B	0.167	0.207	0.131	0.401	12	172	289	11	113	392
AX-11675902	6	30038712	rs9261291	RNF39	[T/C]	5.72E-05	1.50	1.23	1.83	A	0.275	0.317	0.236	0.311	42	215	214	33	178	305
AX-11172896	6	30039364	rs11753382	RNF39	[A/C]	1.38E-06	1.82	1.42	2.32	A	0.162	0.204	0.124	0.749	12	167	289	7	112	391
AX-11133550	6	30046011	rs10947052	TRIM31	[T/C]	1.29E-05	1.70	1.34	2.15	A	0.168	0.206	0.133	0.466	12	171	290	11	115	390
AX-11221691	6	30047301	rs12663534	TRIM31	[A/C]	1.01E-05	1.71	1.35	2.18	B	0.166	0.205	0.131	0.401	11	171	289	11	113	392
AX-11221706	6	30049197	rs12663724	TRIM31	[A/G]	5.03E-06	1.73	1.36	2.19	A	0.172	0.213	0.135	0.545	14	173	285	11	117	386
AX-11184474	6	30057695	rs11968367	TRIM31	[T/C]	3.65E-06	1.76	1.38	2.23	B	0.167	0.208	0.130	0.370	12	172	288	11	112	393
AX-11172759	6	30058777	rs11751554	TRIM31	[A/G]	4.17E-06	1.75	1.38	2.22	B	0.168	0.208	0.131	0.401	13	171	289	11	113	392
AX-11313081	6	30060021	rs17187791	TRIM31	[A/G]	4.90E-06	1.73	1.37	2.20	A	0.171	0.212	0.134	0.510	14	172	286	11	116	387
AX-11643259	6	30060783	rs7753935	TRIM31	[A/C]	4.64E-06	1.73	1.37	2.20	A	0.172	0.212	0.135	0.536	14	173	286	11	117	388
AX-11423789	6	30061037	rs28780077	TRIM31	[A/G]	9.70E-06	1.70	1.34	2.15	A	0.173	0.212	0.137	0.615	14	172	285	11	119	385
AX-11313429	6	30061910	rs17194174	TRIM31	[A/G]	2.64E-06	1.76	1.39	2.23	A	0.173	0.215	0.135	0.541	14	175	283	11	117	387
AX-11423790	6	30063086	rs28780080	TRIM31	[T/C]	7.68E-06	1.71	1.35	2.17	A	0.175	0.216	0.139	0.229	15	169	277	13	115	381
AX-11642367	6	30063733	rs7741418	TRIM31	[T/C]	4.64E-06	1.73	1.37	2.19	A	0.174	0.214	0.136	0.582	13	176	282	11	118	385
AX-11220526	6	30065575	rs1264702	TRIM31	[T/C]	6.01E-06	1.72	1.36	2.18	B	0.172	0.212	0.135	0.541	13	174	285	11	117	387
AX-11674566	6	30066031	rs916570	TRIM31	[A/G]	3.53E-06	1.74	1.38	2.21	B	0.172	0.214	0.135	0.536	14	174	285	11	117	388
AX-11674565	6	30066097	rs916569	TRIM31	[A/G]	3.88E-06	1.74	1.37	2.21	A	0.173	0.214	0.135	0.541	14	174	285	11	117	387
AX-11220519	6	30067834	rs1264696	TRIM31	[A/G]	6.16E-06	1.72	1.36	2.18	A	0.173	0.213	0.136	0.577	14	173	285	11	118	386
AX-11220518	6	30067892	rs1264695	TRIM31	[A/G]	5.58E-06	1.73	1.36	2.19	B	0.172	0.212	0.135	0.545	14	173	286	11	117	386
AX-11142608	6	30069795	rs1108052	TRIM31	[A/C]	6.39E-06	1.72	1.36	2.18	B	0.172	0.212	0.135	0.541	13	173	284	11	117	387
AX-11365449	6	30070178	rs2072110	TRIM31	[T/C]	1.03E-06	1.79	1.42	2.27	B	0.175	0.218	0.135	0.536	14	178	280	11	117	388
AX-11143945	6	30070937	rs1110446	TRIM31	[T/C]	7.26E-07	1.81	1.43	2.30	A	0.173	0.217	0.133	0.466	14	177	281	11	115	390
AX-11507209	6	30171881	rs4476873	TRIM26	[T/C]	4.14E-05	1.69	1.31	2.18	B	0.146	0.180	0.115	0.065	9	152	312	11	96	408
AX-11433079	6	30199016	rs3094138	HLA-L	[T/C]	5.23E-05	1.69	1.31	2.17	B	0.144	0.178	0.114	0.142	9	150	314	10	97	408
AX-11433078	6	30200873	rs3094137	HLA-L	[A/G]	5.59E-05	1.68	1.30	2.16	A	0.146	0.179	0.115	0.176	9	151	311	10	99	407
AX-11106403	6	33032905	rs1042872	HLA-DPA1	[T/C]	8.58E-05	0.66	0.54	0.82	A	0.259	0.219	0.296	0.293	31	143	295	50	204	259
AX-11498323	6	33041226	rs4247257	HLA-DPA1	[T/C]	9.09E-05	0.67	0.54	0.82	B	0.260	0.219	0.297	0.328	29	148	293	50	206	260
AX-11382825	6	33060870	rs2295119	HLA-DPB2	[T/G]	5.10E-05	0.64	0.52	0.80	A	0.225	0.185	0.262	0.369	18	139	315	39	190	283
AX-11677642	6	33083574	rs9277643	HLA-DPB2	[T/C]	6.92E-05	0.65	0.52	0.80	A	0.229	0.189	0.265	0.494	15	147	306	39	194	281
AX-11500162	6	85355752	rs4298310	TBX18	[T/C]	5.06E-05	0.59	0.46	0.76	B	0.148	0.114	0.179	0.004	5	98	370	26	132	356
AX-11111841	6	85394092	rs10498945	TBX18	[A/C]	5.60E-05	0.56	0.43	0.75	B	0.121	0.090	0.150	0.009	2	81	388	19	116	380
AX-12386418	7	19914413	rs10247989	RPL21	[T/C]	5.17E-06	0.66	0.55	0.79	A	0.475	0.422	0.524	0.684	85	229	159	139	261	114
AX-12385752	7	19946542	rs10226787	RPL21	[A/G]	5.17E-06	0.66	0.55	0.79	B	0.475	0.422	0.524	0.684	85	229	159	139	261	114
AX-12640095	7	19955305	rs7780042	RPL21	[T/C]	9.44E-05	1.44	1.20	1.74	B	0.360	0.404	0.319	0.912	81	220	172	52	225	238
AX-12653752	7	19970465	rs886781	RPL21	[T/C]	7.98E-05	1.46	1.21	1.76	B	0.332	0.376	0.292	0.447	65	223	182	47	204	260
AX-12419032	7	46133217	rs11770444	TNS3	[T/C]	3.86E-05	0.66	0.54	0.80	B	0.281	0.237	0.321	0.437	20	180	264	56	213	237
AX-12454562	7	46138472	rs13246216	TNS3	[A/C]	1.40E-05	0.64	0.53	0.79	B	0.285	0.238	0.327	0.476	20	181	263	58	217	234
AX-12501160	7	53924589	rs17559045	HPVC1	[T/G]	4.21E-05	1.62	1.28	2.04	A	0.181	0.218	0.147	0.496	24	157	290	13	125	377
AX-11405796	8	1004029	rs2701903	DLGAP2	[T/C]	9.24E-05	0.70	0.59	0.84	A	0.467	0.422	0.510	0.147	84	231	158	141	239	131
AX-11649473	8	24581685	rs7844557	NEFM	[T/C]	5.89E-05	0.64	0.52	0.80	A	0.225	0.186	0.262	0.273	12	152	309	40	189	285
AX-12599941	8	85070206	rs6473525	RALYL	[A/G]	3.77E-05	1.49	1.23	1.81	A	0.312	0.357	0.271	0.659	67	204	202	36	208	272
AX-12402061	8	106253881	rs10955382	ZFPM2	[A/G]	6.45E-05	0.70	0.58	0.83	A	0.470	0.423	0.513	0.130	88	224	161	127	275	114
AX-11602113	9	16217690	rs7023845	BNC2	[A/G]	6.41E-05	1.79	1.34	2.39	B	0.109	0.138	0.082	0.771	12	107	354	4	77	435
AX-11328062	9	73150873	rs17535963	TRPM3	[T/G]	8.22E-05	0.65	0.52	0.80	B	0.220	0.181	0.255	0.431	14	142	313	30	202	282
AX-11137190	10	71802746	rs10999100	H2AFY2	[A/G]	4.17E-05	0.49	0.34	0.69	A	0.078	0.052	0.101	0.184	2	45	426	8	88	418
AX-11366144	10	112535077	rs2078243	RBM20	[T/C]	5.41E-05	2.72	1.64	4.52	B	0.038	0.056	0.021	0.621	0	53	420	0	22	494
AX-11339056	10	112537734	rs17763100	RBM20	[A/G]	3.31E-05	2.84	1.70	4.75	B	0.038	0.056	0.020	0.636	0	53	420	0	21	492
AX-11351042	11	80516382	rs1894061	RPS28	[A/G]	6.83E-05	1.45	1.21	1.75	B	0.379	0.425	0.337	0.608	87	221	157	54	228	217
AX-11573444	12	48116573	rs6580635	ENDOU	[A/G]	4.55E-05	0.60	0.47	0.77	A	0.166	0.131	0.199	0.068	6	111	354	27	151	337
AX-12522272	12	48117535	rs2239188	ENDOU	[T/G]	4.85E-05	0.61	0.47	0.77	A	0.166	0.131	0.199	0.064	6	111	353	27	150	335
AX-11380221	12	94772742	rs2271979	CCDC41	[T/C]	8.18E-05	0.69	0.57	0.83	A	0.354	0.309	0.394	0.856	31	230	211	81	244	190
AX-11691282	13	91085354	rs9515742	LOC144776	[A/G]	7.61E-05	0.62	0.49	0.79	A	0.174	0.138	0.206	0.200	6	119	348	17	177	318
AX-11525002	13	100473105	rs4772248	CLYBL	[T/C]	2.39E-05	1.77	1.35	2.31	B	0.130	0.164	0.100	0.294	14	127	332	3	97	416
AX-12519941	14	22536360	rs2178789	DAD1	[T/C]	3.90E-06	0.65	0.54	0.78	B	0.410	0.356	0.460	0.972	50	225	182	105	246	145
AX-12575112	14	86551182	rs4577004	GALC	[A/C]	3.35E-05	0.30	0.16	0.55	A	0.032	0.015	0.048	0.424	0	14	456	2	45	464
AX-12406219	16	25784859	rs11074720	HS3ST4	[T/G]	8.64E-05	0.60	0.47	0.78	A	0.152	0.118	0.182	0.552	8	95	366	15	157	342
AX-11502415	16	56573297	rs435651	MT4	[T/G]	3.57E-05	0.68	0.57	0.82	A	0.391	0.344	0.435	0.276	55	213	202	103	240	170
AX-11568472	16	56576608	rs6499839	MT4	[T/C]	2.84E-05	1.69	1.32	2.16	A	0.156	0.192	0.123	0.249	22	137	313	5	117	393
AX-11399723	18	73378887	rs2584466	ZNF516	[T/C]	2.31E-05	1.48	1.23	1.77	A	0.394	0.443	0.349	0.194	99	218	153	56	247	211
AX-12595752	20	6956974	rs6077117	HAO1	[T/C]	5.59E-05	0.68	0.57	0.82	B	0.363	0.317	0.404	0.029	45	208	217	72	271	170
AX-11560229	20	46366923	rs6122609	SULF2	[A/G]	8.93E-05	1.48	1.22	1.80	A	0.283	0.324	0.245	0.604	50	206	216	33	186	296

a Odds ratio of allele A from two-by-two allele frequency table.

Table S2. Association analyses of *HLA-B*, *HLA-C*, *-DRB1*, *-DQB1*, and *-DPB1* alleles

HLA-B	case (2n=832)	%	control (2n=922)	%	Fisher's P-value	OR	95% CI	
07:02	23	2.8	26	2.8	1	0.98	0.53	1.80
13:01	14	1.7	16	1.7	1	0.97	0.44	2.13
15:01	51	6.1	78	8.5	0.06704	0.71	0.48	1.03
15:18	12	1.4	15	1.6	0.847	0.88	0.38	2.04
35:01	65	7.8	75	8.1	0.86	0.96	0.67	1.37
39:01	25	3.0	33	3.6	0.5931	0.83	0.47	1.46
40:01	59	7.1	57	6.2	0.5008	1.16	0.78	1.72
40:02	71	8.5	85	9.2	0.6746	0.92	0.65	1.29
40:06	25	3.0	32	3.5	0.5931	0.86	0.49	1.52
44:03	49	5.9	24	2.6	0.0007086	2.34	1.39	4.03
46:01	62	7.5	56	6.1	0.2538	1.25	0.84	1.85
48:01	17	2.0	23	2.5	0.6315	0.82	0.41	1.61
51:01	81	9.7	83	9.0	0.6227	1.09	0.78	1.52
52:01	149	17.9	165	17.9	1	1.00	0.78	1.29
54:01	62	7.5	74	8.0	0.7208	0.92	0.64	1.33
56:01	18	2.2	7	0.8	0.01507	2.89	1.14	8.23
59:01	14	1.7	18	2.0	0.7235	0.86	0.39	1.84
67:01	10	1.2	17	1.8	0.3333	0.65	0.26	1.51

HLA-C	case (2n=896)	%	control (2n=1004)	%	Fisher's P-value	OR	95% CI	
01:02	175	19.5	184	18.3	0.5187	1.08	0.85	1.37
03:03	128	14.3	154	15.3	0.5608	0.92	0.71	1.20
03:04	121	13.5	142	14.1	0.6904	0.95	0.72	1.24
04:01	23	2.6	38	3.8	0.1519	0.67	0.38	1.16
06:02	6	0.7	6	0.6	1	1.12	0.30	4.21
07:02	82	9.2	102	10.2	0.4848	0.89	0.65	1.22
07:04	11	1.2	10	1.0	0.6655	1.24	0.47	3.26
08:01	40	4.5	64	6.4	0.06971	0.69	0.45	1.05
12:02	157	17.5	167	16.6	0.6252	1.06	0.83	1.36
14:02	66	7.4	59	5.9	0.1957	1.27	0.87	1.87
14:03	52	5.8	28	2.8	0.001255	2.15	1.32	3.56
15:02	27	3.0	34	3.4	0.6967	0.89	0.51	1.53

HLA-DRB1	case (2n=914)	%	control (2n=952)	%	Fisher's P-value	OR	95% CI	
01:01	24	2.6	26	2.7	1	0.96	0.52	1.75
04:01	13	1.4	12	1.3	0.8416	1.13	0.47	2.73
04:03	22	2.4	8	0.8	0.009016	2.91	1.24	7.60
04:05	113	12.4	134	14.1	0.3054	0.86	0.65	1.14
04:06	9	1.0	25	2.6	0.008899	0.37	0.15	0.82
04:10	11	1.2	19	2.0	0.1997	0.60	0.26	1.33
08:02	29	3.2	34	3.6	0.701	0.88	0.52	1.51
08:03	106	11.6	89	9.3	0.1299	1.27	0.93	1.73
09:01	173	18.9	205	21.5	0.1671	0.85	0.67	1.07
11:01	21	2.3	15	1.6	0.313	1.47	0.72	3.08
12:01	44	4.8	33	3.5	0.1626	1.41	0.87	2.31
12:02	23	2.5	26	2.7	0.885	0.92	0.50	1.69
13:02	25	2.7	13	1.4	0.04794	2.03	0.99	4.35
14:03	6	0.7	5	0.5	0.7697	1.25	0.32	5.20
14:05	24	2.6	14	1.5	0.1002	1.81	0.89	3.80
14:54	34	3.7	34	3.6	0.902	1.04	0.62	1.75
15:01	64	7.0	82	8.6	0.1974	0.80	0.56	1.14
15:02	163	17.8	163	17.1	0.7146	1.05	0.82	1.34

HLA-DQB1	case (2n=930)	%	control (2n=1012)	%	Fisher's P-value	OR	95% CI	
03:01	105	11.3	94	9.3	0.155	1.24	0.92	1.69
03:02	48	5.2	65	6.4	0.2456	0.79	0.53	1.18
03:03	190	20.4	224	22.1	0.3751	0.90	0.72	1.13

04:01	114	12.3	141	13.9	0.2826	0.86	0.66	1.13
04:02	24	2.6	33	3.3	0.4205	0.79	0.44	1.38
05:01	29	3.1	33	3.3	0.8977	0.95	0.55	1.64
05:02	18	1.9	28	2.8	0.2367	0.69	0.36	1.31
05:03	47	5.1	44	4.3	0.5193	1.17	0.75	1.83
06:01	270	29.0	254	25.1	0.05202	1.22	0.99	1.50
06:02	57	6.1	80	7.9	0.1323	0.76	0.53	1.10
06:04	24	2.6	12	1.2	0.0278	2.21	1.05	4.87

**HLA-DPB1**	case (2n=926)	%	control (2n=1022)	%	Fisher's P-value	OR	95% CI	
**02:01**	144	15.6	243	23.8	**5.23E-06**	**0.59**	0.47	0.75
02:02	26	2.8	27	2.6	0.8894	1.06	0.59	1.91
03:01	52	5.6	51	5.0	0.5448	1.13	0.75	1.72
04:01	22	2.4	14	1.4	0.1287	1.75	0.85	3.73
04:02	47	5.1	46	4.5	0.5953	1.13	0.73	1.76
05:01	461	49.8	467	45.7	0.07649	1.18	0.98	1.41
09:01	146	15.8	143	14.0	0.2785	1.15	0.89	1.49
13:01	13	1.4	16	1.6	0.8524	0.90	0.39	2.00
14:01	11	1.2	9	0.9	0.5106	1.35	0.51	3.71

Table S3. Demographic details and number of samples analyzed in GWAS and replication study

		GWAS	Replication 1	Replication 2	Replication 3
population		Japanese	Japanese	Hong Kong Chinese	Thai
number of samples	total	989	767	281	383
	HCC	473	153	94	185
	CH, ASC	516	614	187	198
age (sd)	HCC	61.0 (10.34)	61.3 (8.36)	58.0 (10.53)	58.4 (12.02)
	CH, ASC	56.0 (12.63)	55.1 (10.15)	56. 9 (8.31)	46.4 (11.07)
gender (male %)	HCC	390 (82.8%)	117 (76.5%)	80 (85.1%)	151 (82.1%)
	CH, ASC	265 (54.6%)	298 (48.5)	159 (85.0%)	131 (66.2%)

Abbreviation: HCC, Hepatocellular Carcinoma; CH, Chronic Hepatitis; ASC, Asymptomatic carrier.
